# The impact of strain, constraints, and morality on different cyberbullying roles: A partial test of Agnew’s general strain theory

**DOI:** 10.3389/fpsyg.2022.980669

**Published:** 2022-10-17

**Authors:** Wanqi Li, Huaxin Peng

**Affiliations:** School of Media and Communication, Shenzhen University, Shenzhen, Guangdong, China

**Keywords:** GST, negative emotion, self-control, protective variables, cyberbullying perpetration, cyberbullying victimization, China

## Abstract

The strain has been well studied in traditional bullying, and recent research has begun to explore it in cyberbullying behavior. Drawing from General Strain Theory, the current study empirically examined the relationship between strain and the cyberbullying behavior of perpetrators and bully-victims, respectively. Meanwhile, this study also considered the influences of the protective variables (e.g., constraints and morality) on the strain, which may potentially reduce the risks of participating in cyberbullying. The sample comprised 928 Chinese internet users (Male = 490, Female = 438) aged between 16 to 50. We identified the prevalence of cyberbullying in China, in which the percentages of cyberbullying perpetrators, cyberbullying victims, and cyber bully–victims were 23.40, 23.20, and 37.40%, respectively. This study mentioned a noteworthy phenomenon: cyberbullying victims quickly became cyberbullying perpetrators when they were cyberbullied. Secondly, according to the binary logistic regression, we hold that the strain was significantly related to cyberbullying behaviors, as individuals with low levels of self-control showed a higher tendency to participate in cyberbullying. As for protective factors, the results showed that constraints and morality can reduce the negative consequences of strain and then against cyberbullying, exploring the possibilities of using constraints and morality as variables to decrease strain and prevent cyberbullying. Thus, the unique values of this study are using the GST theory to investigate the empirical link between strain and cyberbullying between different roles in a new cultural and social background, demonstrating that negative emotion and low self-control had influences on strain and cyberbullying behaviors. Meanwhile, this study also contributes by discussing the implications for future research and practicing efforts targeting how to decrease the risks of cyberbullying engagement, for example, we suggest that the prevention and intervention of cyberbullying should adopt a cross-sectoral response to help individuals to view cyberbullying, vent dissatisfaction and relieve strain in the right way.

## Introduction

Cyberbullying has become a more pervasive delinquent behavior with negative consequences along with advances in online communication technology. In the previous studies, researchers have extensively used traditional bullying elements, such as repetition, intention to harm, and imbalance of power, to define cyberbullying (e.g., Olweus and Limber, 2018; Baldry et al., 2019; Paciello et al., 2020), regarding cyberbullying as a repeated and aggressive behavior, based on a power imbalance, to intentionally hurt or embarrass a target through the use of electronic communication tools (Patchin and Hinduja, 2012; Ira-Katharina and Petermann, 2018; Olweus and Limber, 2018; Clark and Bussey, 2020). However, cyberbullying has some different characteristics (e.g., publicity, anonymity, and the 24/7 nature) from traditional bullying (Peterson and Densley, 2017; Li and Hesketh, 2021) and cyberbullying participants also displayed their differences from participants in traditional bullying (Camerini et al., 2020).

### Cyberbullying and traditional bullying

To differentiate between cyberbullying and traditional bullying, previous studies have mainly discussed the applicability of repetition and the power imbalances in cyberbullying (e.g., Bauman et al., 2013; Slonje et al., 2013). Specifically, the idea of repetition within cyberbullying may have two directions: offensive contents directly posted by one perpetrator repeatedly and offensive contents indirectly distributed by other people repeatedly (Slonje et al., 2013). These two forms are the cyberbullying behavior experienced many times by the victim, but the perpetrator may not be the initial one (Ibrahim, 2022). Meanwhile, the term of power imbalance also has new features in cyberspace. In the traditional bullying behavior, Olweus (1994) described the power as a physical strength or a social rank between perpetrators and victims. In other words, the victim in the traditional bullying was the weak part because they had physical and psychological weakness compared with the perpetrators (Olweus, 1994). However, physical strength is not necessary for cyberbullying perpetration, and thus, forms of power imbalance in the cyberbullying behavior were affected by virtue of participants’ numbers or were attributed to an imbalance in technological skills between the parties (Slonje et al., 2013). Specifically, the more advanced technological skills or media expertise the cyberbullying perpetrators have, the greater the possibility that their cyber victims would be cyberbullied, and in turn, the cyberbullying victims can escape cyberbullying if they have good technical ability to “block” the perpetrators to prevent harmful consequences (Slonje et al., 2013). Furthermore, the publicity and the 24/7 nature of cyberspace also showed different features between cyberbullying victims and perpetrators (Cassidy et al., 2018). For example, cyberspace is an open and anonymous environment that may provide a more complex situation for cyberbullying than traditional face-to-face bullying issues (Clark and Bussey, 2020).

In addition to the features of cyberbullying and traditional bullying, the concept of cyberbullying roles is also necessary to clarify. Salmivalli (1999) was the first to distinguish participant roles in traditional bullying, namely, the victim, the perpetrator (bully), the bully’s assistant (who encourages the bullying), the bully’s reinforcer, the victim’s defender, and the bystander. These were later applied to cyberbullying, and the core roles in cyberbullying behavior are perpetrators, victims, and bystanders (Musharraf and Anis-ul-Haque, 2018; Moxey and Bussey, 2019). Importantly, the cyberbullying perpetrator and the victim are interchangeable, in which victims used cyberbullying as a way to protect themselves, and thus, plus a new role, the cyber bully–victim, who are both cyberbullying victims and cyberbullying perpetrators (McInroy and Mishna, 2017; Espino et al., 2022). Regarding cyberbullying perpetrators and victims, researchers agreed that perpetration and victimization co-occur far more frequently in cyberspace than in the offline setting, because offensive messages spread quickly online (Musharraf and Anis-ul-Haque, 2018; Clark and Bussey, 2020; Parti et al., 2022). Meanwhile, Espino et al. (2022) showed that if cyberbullying victims have experienced frequent aggression, they are more likely to perpetrate cyberbullying. These points show that we shall consider the challenges when the aspects of traditional bullying features directly applied in cyberbullying (Slonje et al., 2013), and cyberbullying is an interaction that could be predicted and prevented through interpersonal relationships (Varela et al., 2018; Mehari and Basu, 2022). Therefore, this study defined cyberbullying as a new form of deliberate, aggressive behavior toward an individual or a group of individuals through digital devices at anytime and from anywhere.

### Prevalence and forms of cyberbullying

During the early phases of research on cyberbullying, studies mainly focused on cyberbullying among adolescents and youths (e.g., Hong et al., 2017; Chu et al., 2018; Li and Hesketh, 2021), yet, in the last few decades, researchers agreed that cyberbullying can be a group or an individual behavior and then moved their sights on adult targets (e.g., Barlińska et al., 2015; Ouvrein et al., 2017; Saengprang and Gadavanij, 2021; Giumetti and Kowalski, 2022; Rudnicki et al., 2022). In Moretti and Herkovits’ (2021) study, they used meta-ethnography to summarize the findings from 33 studies, concluding that the prevalence of cyberbullying among respondents (including teenagers, youths, and adults) varied from 10 to 40% in different countries (e.g., Netherlands, Sweden, United Kingdom, United States, Australia, and Thailand). In China, there are no accurate official figures on how many adolescents and adults experience cyberbullying per year, but different studies have indicated that the prevalence of cyberbullying ranged from 3.00 to 69.00% (e.g., Chan and Wong, 2015; Chen and Chen, 2020). Meanwhile, the popularity of both cyberbullying perpetrators and victims have grown in recent years in various studies. For example, Camerini et al. (2020) identified 76 original longitudinal studies published between 2007 and 2017, summarizing that prevalence rates for cyberbullying perpetration varied between 5.30 and 66.20%, and cyberbullying victimization ranged from 1.90 to 84.00%. Cyberbullying has undoubtedly become a serious social problem along with the flourishing of social media sites.

In addition to the prevalence of cyberbullying, there is increasing concerns over the forms and consequences of cyberbullying on an international scale (Yuchang et al., 2019; Polanin et al., 2021). The most widely used cyberbullying taxonomy is developed by Willard (2007) who identified cyberbullying behaviors as photo and video clip bullying, flaming and threatening with vulgar language, excluding someone from an online group, posting material that contains private information, spreading rumors and denigration, cyberstalking and cyber threats, and online harassment. These behaviors can cause severe psychological problems and health-related issues, such as anxiety, depression, isolation, lower self-esteem, stress, negative moods, violent behavior, and even suicide (Smith, 2012; Berne et al., 2019; Pittaro, 2020; Barlett et al., 2021; Li and Hesketh, 2021; Marzano, 2022). Significantly, some researchers (Campbell et al., 2012; Hellfeldt et al., 2020; Marzano, 2022) agreed that cyberbullying victims may suffer from more severe mental health problems than traditional bullying victims for two reasons. On the one hand, cyberbullying perpetrators can easily use pseudonyms, and more importantly, cyberspace allows them to anonymously inflict harm on victims irrespective of time and location (Lowry et al., 2016). On the other hand, cyberbullying victims may not only suffer massive abusive or humiliating communications from the perpetrator, but also may experience cyberbullying repeatedly by “bystanders” (Extremera et al., 2018; Gafney et al., 2019; Marzano, 2022). Under such circumstances, the offensive communications become “snowballs” which were out of the control and made cyberbullying victims experience a sharp decline in self-esteem and more severe depression, eventually pushing them to cyberbullying engagement (Chen, 2016; Kwan et al., 2020). However, the finding of cyberbullying consequences to victims or perpetrators might be different in various studies due to the different research samples (Ira-Katharina and Petermann, 2018).

### Application of general strain theory and cyberbullying behavior

Considering cyberbullying issues, several studies have used the General Strain Theory (GST) to explain that cyberbullying can result from strain caused by weak social control (Agnew, 2006; Park and Metcalfe, 2020; Quintana-Orts et al., 2020). Agnew’s (1992) general strain theory (GST) was originally an inductive framework put forward by Merton (1949). This theory states that individuals who experience (1) failure to achieve positively valued goals, (2) loss of positively valued stimuli (for example, parental loss), and (3) the presence of negative stimuli (for example, negative emotions or emotional abuse), were under strain or stress. According to GST, both externalizing responses (i.e., acts that harm others either *via* violence) and internalizing responses (i.e., acts committed against themselves, such as substance use and deliberate self-harm) were found to be associated with various forms of strain (Guo, 2021). In the previous studies, the associations between strain and traditional bullying have been well studied (e.g., Moon and Jang, 2014; Espino et al., 2022; Strohacker et al., 2022). For example, Agnew (2011) found that individual with high levels of strain is more likely to engage in the traditional bullying behavior; Ganem (2010) stated that negative emotionality affects the relationship between strain and offending; Cochran (2015) have used self-domain, family domain, school domain, peer domain, and deviant motivations as main variables in testing strain and traditional bullying; Wooten (2021) investigated that strain leads to anger, which in turn leads to delinquency.

More recently, with a higher incidence of cyberbullying in the online environment, existing research pointed out that cyberbullying might also be an outcome of strain, and mainly analyzed what and how variables can influence the relations between strain and cyberbullying (Brailovskaia et al., 2018; Lianos and McGrath, 2018; Shin and Kim, 2022; Wilson and Seigfried-Spellar, 2022). For cyberbullying research, studies have provided a standard view, stating that engagement in cyberbullying was the result of physical, emotional, or psychological strain (Jang et al., 2014; Lianos and McGrath, 2018), and participation in cyberbullying through strain-based factors (e.g., receiving bad grades, being treated unfairly by someone, victimization, and relationship issues) was a response to strain (Patchin and Hinduja, 2011). Specifically, Cui and To (2021) used a school-based multistage random sample of 1,666 children in Grades 4 to 9 in China (Nanjing and Guangzhou), indicating that the experience of cyberbullying victimization, which can be a deviant motivation, has frequently been recognized as a reason of strain which can predict cyberbullying perpetration. Consequently, this study mainly focuses on how do the negative emotion and the low self-control affect cyberbullying between different roles in the Chinese context.

#### Associations between negative emotion, strain, and cyberbullying roles

In GST research, Agnew (1992) stated that negative emotion was the most critical emotional reaction, and in 2001, Agnew suggested that individuals who suffered from strains may experience negative emotions and were more at risk of engaging in bullying, criminal, or deviant behavior. Some researchers have developed the idea and stated that negative emotions were an outcome of strain and different types of strain might produce different negative emotions (Lianos and McGrath, 2018; Wang and Jiang, 2021). In this study, we anticipate negative emotions can also lead to strain and then result in cyberbullying. Given that different cyberbullying roles may cause different degrees of negative emotions, the roles played by cyberbullying-involved participants may have different risks of delinquency (Guo, 2021). On this view, Wang and Ge (2021) explored the associations between psychological distress among adolescents and their cyberbullying behaviors. They used a sample of 607 adolescents aged from 13 to 18 years to investigate that online and offline victimization positively associated with depression, which, in turn, predicted higher possibilities of cyberbullying perpetration (Wang and Ge, 2021). Meanwhile, Giumetti et al. (2022) randomly selected university students (final year) in the eastern United States to find that cyberbullying victims were more related to anxiety and depression and had risks to participate in delinquent behaviors. Furthermore, in the Chinese context, Zhang et al. (2020) examined the longitudinal associations between neuroticism, depression and cyberbullying (perpetration and victimization) in China through multiple measurements. The sample contained 3,961 Chinese early adolescents (M = 10.85 years), and Zhang et al. (2020) found that depression predicted both subsequent cyberbullying (perpetration and victimization). Interestingly, compared with pure perpetrators of cyberbullying, victims of cyberbullying reported more negative emotions, such as depression, anxiety, and anger (Keith, 2018; Lianos and McGrath, 2018). Therefore, greater aggressiveness of victimization maybe more closely linked to perpetration, because the experience of cyberbullying victimization was the most important source of negative emotions and strain which can lead to offensive behavior (Bayraktar et al., 2015; Brailovskaia et al., 2018; Lee et al., 2021). Overall, the negative emotion may in turn impose a greater strain on individuals and ultimately lead them to adverse behavior (Coelho and Romão, 2018).

#### Associations between self-control, strain, and cyberbullying roles

In addition to negative emotions, self-control is also an important element when we consider the associations between strain and cyberbullying. Self-control is people’s abilities (e.g., problem-solving and attentional control) to regulate themselves which can pursue a more positive response to deviance and reduce the harmful effects of bullying behavior (Wang and Ge, 2021). As Agnew (2001) stated, self-control may be the most potent factor affecting the relationship between strain and aggressive behavior. For the cyberbullying research, Gottfredson and Hirschi (1990) argued that individuals with low self-control were more likely to engage in antisocial behaviors (e.g., alcohol consumption, bullying, and sexually promiscuous behavior), because they could not inhibit their unwanted behaviour. Meanwhile, Li et al. (2016) used a sample of middle and high school students in Kentucky, utilizing how low self-control (e.g., impulsive, insensitive, risk-taking, and short-sighted) influence on the engagement in cyberbullying. Li et al. (2016) postulated that low self-control directly increases the risk of strain and then allows cyberbullying to arise. Meanwhile, Lianos and McGrath (2018) selected a sample that contained 320 Internet-active young adults to prove a correlation between self-control and cyberbullying, investigating that low self-control and higher levels of strain related to cyberbullying perpetration. Furthermore, Choi et al. (2022) tested a relationship between self-control and deviant behavior among a sample of 1,091 South Korean adolescents, stating that low self-control was a significant positive predictor of cyber perpetration.

As above, we could infer that the negative emotion or the weakened self-control easily strengthened one’s feelings of strain, drove individuals to break rules, and brought about a higher incidence of cyberbullying. Notably, in China, a remarkable feature of cyberbullying is that most internet users regard cyberbullying as a way to express emotions (Bao et al., 2014; Xu, 2021). Specifically, when individuals have negative emotions, they may readily have strain and then use aggressive language toward others (Ye et al., 2021; He et al., 2022), which may spark aggressive expressions toward themselves. Therefore, emotion plays an essential role in cyberbullying, especially for cyberbullying victims (Keith, 2018). Meanwhile, most internet users regard cyberbullying as a herd mentality (Xu, 2021). Under this circumstance, when individuals engage in cyberbullying in China, they may identify cyberbullying as group behavior and subjectively reduce the impact of their deviant actions on the victim (Willis et al., 2019). At this point, the influence of self-control on cyberbullying may decrease, making the individual believe that *everyone is doing this, and so can I* (Piquero et al., 2016; Ye et al., 2021).

#### Associations between constraints, morality, strain, and cyberbullying roles

Considering the risk factors of strain and cyberbullying, several studies have attempte to examine the protective factors to prevent cyberbullying, stating that individuals mainly cope with strain through problem-focused and emotion-focused coping strategies (Raskauskas and Huynh, 2015; Xue et al., 2022). Empirical research on the effects of constraints and the influences of morality on strain are worth noting, to explore how they decrease strain and then decrease the risks of engaging in cyberbullying.

Self-control and constraints are often used interchangeably in the GST model (Agnew, 2013), but when considering how they affect cyberbullying, the differences between self-control and constraints are that self-control is a stable personality trait that develops in the context of insufficient socialization in early childhood Gottfredson and Hirschi (1990), but various life domains can act as sources of constraints to make people regulate their behavior at any stage (Kabiri et al., 2020). Agnew (2005) defined constraints as factors that deter, inhibit, or dissuade individuals from engaging in criminal behavior. Constraints contains internal control (referring to moral values and attitudes toward the law and social norms), informal control (referring to certainty/severity sanctions or formal/informal punishments applied by others), and formal control (referring to various constraints that have been established against crime; Agnew et al., 2000; Bao et al., 2014; Cochran, 2015; Choi and Kruis, 2019). Following the above views, Zhang et al. (2012) used longitudinal data of Paternoster’s Youths and Deterrence (1979–1981) to examine whether family factors promote delinquency by diminishing constraints against delinquency, stating that those under close parental supervision are more likely to perceive constraints against delinquency. Thus, the likelihood of engagement in aggressive behaviors decreases. Furthermore, some researchers stated that peers’ behavior and views are also significantly associated with cyberbullying perpetration (Hong et al., 2017; Paez, 2018; Kabiri et al., 2020). Specifically, Shim and Shin (2016) focuses on the role played by peer-group pressure. They used a randomly selected students from high schools and junior high schools in South Korea to state that cyberbullying frequently occurs when peers endorse similar behaviors, which means an individual may exhibit a high level of cyberbullying perpetration if he/she engages in aggressive behavior frequently. Accordingly, strains may pressure one into deviance when constraints are low (Cochran, 2015).

In addition to constraints, morality, which is a person’s cognition of regulating their behaviors and actions in ways that conform to their moral values (Aquino et al., 2009), is valuable to discuss in the cyberbullying research. A standard view in previous studies is that morality significantly prevents cyberbullying perpetration (Yang et al., 2018). Aquino et al. (2009) used friendliness, generosity, and helpfulness as moral qualities to explore how morality affects aggressive actions, stating that a person with a high moral identity is more likely to regulate their behavior. The major influences of morality on cyberbullying remind individuals to realize moral values and favor their positive interactions with others (Shadmanfaat et al., 2019).

In the previous studies, Zhao et al. (2022) used a total of 620 high school participants from southeast China to investigate the association between morality and cyberbullying behavior. They found that morality was negatively associated with cyberbullying behavior. Although this finding showed the protective functions of morality on cyberbullying, some questions still remain. For example, recently in China, the government and social media operators have established numerous rules and standards to address negative online behaviors (Flew et al., 2019). Nevertheless, the effectiveness of these socialization rules is limited in regulating cyberbullying because they do not have a clear definition and regulatory means (Li, 2020). Meanwhile, cyberbullying in China often forms group behavior, and perpetrators may believe they only play a small role in the offensive behavior (Yang et al., 2018). Under this circumstance, the influence of morality, as well as constraint, on strain might be weakened, which may compel one to violate social rules and give rise to cyberbullying behavior (Cochran, 2015). However, a few studies studied the associations between constraints, morality, strain, and cyberbullying engagements in the Chinese cultural context. Accordingly, this study aims to explain how constraints and morality can influence strain, with a special focus on the links between different cyberbullying roles.

### The aims of this study

Despite ample research conducted to test general strain theory’s applicability to understanding cyberbullying, can introducing certain protective variables with features of the online environment and cultural background in China strengthen or clarify the association between strain and cyberbullying roles remains a question unanswered. Firstly, a few studies attempted to explore the pathways through which cyberbullying roles link with strain within the integrated model of GST, especially in the Chinese context. Secondly, direct and indirect relationships between protective factors and strain might depend upon specific cyberbullying roles (e.g., perpetrator, victim, and bully-victim), which should be carefully considered. Therefore, it is necessary to examine whether core elements originally developed to explain delinquency can adequately explain the pervasive behavior of cyberbullying in China.

To partly close the research gaps mentioned, this study focused on the risk factors that would affect strain and cyberbullying behavior, mainly choosing cyberbullying perpetrators and bully-victims, as both of them involve perpetrators, to clarify interactive mechanisms of risk and protective factors (constraints and morality) of cyberbullying behavior. Firstly, we questioned how do negative emotions and low self-control influence the effects of strain on cyberbullying perpetration in China? We therefore hypothesized that (H1) individuals with higher levels of negative emotions are more likely to have higher levels of strain and, in turn, they are more likely to become cyberbullying perpetrators (H1a) or cyber bully-victims (H1b); and (H2) individuals with lower levels of self-control are more likely to have high levels of strain and accordingly, they are more likely to become cyberbullying perpetrators (H2a) or cyber bully-victims (H2b). Secondly, we directly explored the empirical link between strain and cyberbullying, proposing the hypothesis that (H3) strain has significant effects on cyberbullying perpetrators’ (H3a) or cyber bully-victims’ (H3b) engagements in cyberbullying. Thirdly, considering the directly or indirectly influences of constraints and morality on decreasing the risks of strain and preventing cyberbullying, this study proposed the hypotheses that: (H4) constraints have protective influences between strain and cyberbullying that those having higher levels of constraints, the likelihood of engagement in cyberbullying behaviors decreases; and (H5) morality has protective influences between strain and cyberbullying that those experiencing lower levels of morality, the likelihood of engagement in cyberbullying behaviors increase.

## Materials and methods

### Participants

There were 490 males and 438 females from 27 provinces (e.g., Guangdong, Sichuan, and Hubei), municipalities (e.g., Beijing and Chongqing) and autonomous regions (e.g., the Guangxi Zhuang Autonomous Region) in mainland China who filled the questionnaire. About one-third of the participants were aged 16–17 (29.80%). Another 25.40% of participants (236) aged 18 to 25, and 26.10% (242) of them were between 26 and 30. Participants aged from 31 to 40 account for 11.70, and 6.90% of participants aged from 41 to 50. There were no participants aged over 50. Meanwhile, more than half had an associate degree (32.40%) or bachelor’s degree (29.40%), and 17.70% had a junior high school education and 15.80% had a high school education. There were 4.60% of participants had a postgraduate degree. For the use of social media, Sina Weibo was the most frequently used social media (61.40%), followed by Toutiao at 45.20% and Zhihu (similar to Quora) at 35.70%. Furthermore, 31.50% of participants used social media one to 2 h per day, and 29.20% spent 3–4 h on social media per day, ranking second. In addition, 33.50% of the participants had used social media for 1–3 years, while 290 (31.30%) participants had used social media for less than 1 year. For the attitude toward cyberbullying, more than half of the participants (62.80%) got frightened of cyberbullying. At the same time, 41.10% of the participants regarded cyberbullying as a way of expressing emotions, and 48.90% thought cyberbullying could be used to express anger.

### Materials

This study analyzed cyberbullying in general, including both adult and young participants. Cyberbullying Behavior Perpetration (CBP) and Cyberbullying Behavior Victimization (CBV) were separately measured using 12 single-choice questions adapted from Chen and Chen’s (2020) study. The participants were first asked to answer two single-choice questions - (1) Have you been cyberbullied in the last year (yes/no)? and (2) Have you participated in cyberbullying in the last year (yes/no)? If the answer was “yes,” they would be invited to choose at least one and at most three options related to their experiences. There is a corresponding logical relationship between single-choice questions and CBP/CBV questions. In other words, if the participants answered “no” to the single-choice question, they would skip the following CBP/CBV questions. The CBP questions included “I harass others with frequently posting information,” “I attacked and insulted others,” “I attacked others with sexual words,” and “I cursed others,” “I squeezed others out of online communities,” “I threatened or intimidated others,” “I slandered others online,” “I called others’ nicknames,” “I breached other people’s privacy online (such as posting addresses, photos, and phone numbers online),” “I coined fake images, videos and audios of others and posted online,” “I attacked others’ family members, friends or important people online,” and “I cursed others’ family members, friends or significant ones online.” The CBV experiences were the corresponding CBP questions, for example, including “I was attacked and insulted,” “I was attacked with sexual words,” and “I was cursed.”

For identifying cyberbullying roles, if participant’s answer was “yes” for the first question but was “no” for the second question, they were labeled as 1 = “cyberbullying victim”; if their answer was “no” for the first question but was “yes” for the second question, they were labeled as 2 = “cyberbullying perpetrators”; if their answers of the two questions were “yes,” they were labeled as 3 = “cyber bully-victims”; and if the answers for the two questions were “no,” they were labeled as 0 = “non-involved.” These four categories were also consistent with results from previous studies (Hellfeldt et al., 2020), and these categories also help to code the CBP and CBV as the binary variables for further analyses.

The strain was tested by different dimensions of peer strain, academic or work strain, and financial strain, with a total of 13 items. Peer strain and academic/work strain were measured using items employed by Jang et al. (2014), and financial strain was measured using the questions developed by Lianos and McGrath (2018). Participants were invited to indicate their agreement with the statements on a five-point rating scale, ranging from 1 (strongly disagree) to 5 (strongly agree). The peer strain scale includes sample items (1) “I get stressed by infertile communication with peers,” and (2) “I get stressed by disputes with peers.” The academic or work strain scale includes sample questions: (1) “I get stressed by poor performances in studying or working,” and (2) “I get stressed by preparation for college or occupation.” The financial strain scale includes sample items: (1) “I get stressed by lack of money,” and (2) “I get stressed by not being able to get goods that I want.” The Cronbach’s alpha (α) of the strain scale is 0.97.

Self-control was adapted from the Chinese version of the self-control scale (Tan and Guo, 2008). Items were measured on a five-point scale (1 = strongly disagree to 5 = strongly agree). The questions include (1) “I do certain things that are bad for me if they are fun”; (2) “I lose my temper whenever I get angry”; and (3) “I enjoy teasing and harassing other people.” The items were reversely coded, with a lower score indicating a higher level of self-control. A higher score indicates a higher level of strain. The Cronbach’s alpha (α) is 0.89.

Negative emotion was adapted from Luo et al. (2011) and Wang et al. (2019), including two subscales of temperamental traits anger and depression. Negative emotion uses the five-point scale (1 = strongly disagree to 5 = strongly agree). Sample questions include (1) “I have a fiery temper”; (2) “I feel fearful or anxious”; (3) “I always feel sad or depressed”; (4) “I feel nobody loves me”; and (5) “I have sudden changes in mood or feeling.” A higher score indicates a higher level of negative emotions. The Cronbach’s alpha (α) is 0.96.

The constraint is a 6-item measure adapted from Cochran’s (2015) survey (Appendix: Terms of constraint). The scale includes three dimensions: internal control (2 items), informal control (2 items), and formal control (2 items). Responses to each item were measured on a 4-point Likert scale (1 = definitely would not/no problem at all to 4 = definitely would/huge problem). The Cronbach’s alpha (α) is 0.89.

Morality was measured using items developed by Aquino and Reed (2002). Responses ranged from 1 (strongly disagree) to 5 (strongly agree). The questions include (1) “it is important for me to be a kind and warm person”; and (2) “I strongly desire to have good characteristics.” A higher score indicates a higher level of morality. The Cronbach’s alpha (α) is 0.96.

Finally, gender, age, education, frequently used social media, time spent on social media, years of using social media, and attitude(s) toward cyberbullying are used for the significance test of difference.

### Research procedure

This study used a convenience sample hosted on a survey-hosting website (www.wjx.cn) to test hypotheses. The online survey link was shared through WeChat and Sina Weibo from January 2022 to March 2022, until sufficient samples were collected. To maintain confidentiality, voluntary consent was shown on the first page of the questionnaire, including the introduction of the project, research aims, and informed consent. Especially, for the minors (aged 16 and 17) who were willing to participate in the survey, the electronic parental consent (including the introduction of the project, research aims and precautions) was sent to the participants’ parents. Meanwhile, the anonymity of the respondents and the confidentiality of their responses were strictly guaranteed. This questionnaire contains a test question to improve sample authenticity. The authors’ affiliated institution approved the survey.

Throughout the research, the current study used survey data that covered multiple age groups from China to address research questions, because cyberbullying has become a social problem for both young and adult individuals from various demographic groups. However, participants under 16 were excluded because this group of participants may not have enough knowledge to identify cyberbullying and other issues involved in the convenient survey independently. All participants were told that there were no right or wrong answers, and they could stop the questionnaire anytime. A total of 1,031 questionnaires were distributed, with 928 valid answers received, and 103 (9.91%) invalid questionnaires returned, including questionnaires with incomplete responses and errors in the test question.

### Validity analysis and factor analysis

We tested the exact 5-factor structures obtained by the exploratory factor analysis (EFA) in the SPSS version 26.0. Firstly, Kaiser-Meyer-Olkin value was 0.94 and Bartlett’s test of sphericity (*χ*^2^/*df* = 10.81, *p* = 0.00) was significant, each indicating a rationale for performing EFA. The number of factors to extract was based on Eigenvalues greater than 1.0 and the varimax-rotation method. Examination of the scree plot suggested that five factors were most appropriate. Accordingly, the results yielded five principal components with Eigenvalues of 9.98, 6.91, 5.37, 3.95, and 3.36, respectively. Among the 40 items, all the factor loadings were over.50 (ranged between 0.65 and.88) and communalities of greater than.40. None of the items had substantial cross-loading on other factors. The five factors accounted for 73.91% of the variance in scores. The indices suggested that each item substantially contributed to the factor at very good and excellent levels.

Factor one related to peer strain, academic strain and economic strain and was named as the strain factor. Factor two consisted of items related to personal qualities and perceptions of behavior, which was labeled the morality factor. Factor three was named the negative emotion factor because all of its items represented the personal negative feelings such as anger, depression and unpleasant moods. Factor four represented the internal, external and formal regulations, and was named constraints. Factor five related to the ability to regulate the behavior and was named as self-control.

The confirmatory factor analyses (CFA) were completed by using the SPSSPRO to test the convergent validity and discriminant validity. The following multiple fit indices were used: the relative chi-square ratio (*χ*^2^/*df* < 5, Wheaton et al., 1977), comparative fit index (CFI ≥ 0.95, Hu & Bentler, 1999), the root mean square error of approximation (RMSEA ≤0.06; Hu & Bentler, 1999), the goodness-of-fit index (GFI ≥ 0.80; Muis et al., 2007), the Tucker-Lewis Index (TLI ≥ 0.90; Wongpakaran et al., 2018), the root mean square residual (RMR; Moss, 2009), the normed fit index (NFI ≥ 0.90; Wongpakaran et al., 2018), and non-normed fit index (NNFI ≥0.90; Hu & Bentler, 1999). Meanwhile, values as high as.08 indicated an acceptable fit (Hu & Bentler, 1999; Wongpakaran et al., 2018), and Bentler (1995) suggested that CFI value above.90 was considered indicative of minimally acceptable model fit. Accordingly, the indices provided an acceptable model fit in the current study, for example, *χ*^2^/*df* = 1.89, CFI = 0.92, RMSEA = 0.06, GFI = 0.85, TLI = 0.92, RMR = 0.07, NFI = 0.85, and NNFI = 0.92. Although the index of NFI did not meet the standard that it was sensitive to sample size (Hooper et al., 2008), the values of CFI, TLI and NNFI all exceeded 0.90. Wongpakaran et al. (2018) also stated that NFI or TLI exceeded.09 could indicate an acceptable fit in practice. In addition, the values of AVE ranged from 0.56 to 0.77 (>0.50; Mokhtar et al., 2018), and the values of CR were all over 0.7 (Mokhtar et al., 2018), supporting a convergent validity. Furthermore, the square roots of the AVE that were greater than the intercorrelations between the variables, appearing to have a good discriminant validity (Fornell and Larcker, 1981).

### Statistical analyses

SPSS version 26.0 (Armonk, NY: IBM Corp.) was used to obtain descriptive statistics and the result of correlation analysis. The absolute value of the Skewness of all items in the scales used in this study is below 3, and the absolute value of the Kurtosis is below 10, indicating that all scales conform to a normal distribution. Therefore, the main data analysis included three parts. Firstly, descriptive analysis introduced the general information on cyberbullying perpetration and cyberbullying victimization for the total sample shown in our survey, and the t-test was used to examine the significance test of difference between gender, age, education, frequency of using social media, the years of using social media and cyberbullying roles. Secondly, the binary logistic regression analysis (SPSS v. 26.0) was used to examine hypotheses, because the dependent variables (cyberbullying perpetrators (CB1) and cyber bully-victims (CB2)) were binary variables in this study (Maroof, 2012). Specifically, the binary logistic regression tested the relationship among negative emotion, self-control, strain and cyberbullying. Based on the average scores for negative emotion (mean = 3.18) and self-control (mean = 3.10), participants were categorized as low negative emotion participants (<3.18), high negative emotion participants (>3.18), low self-control participants (<3.10), and high self-control participants (>3.10). We will run a binary logistic regression for the different groups. Therefore, the binary logistic regression attempts to predict the influences of negative emotion and low self-control on strain and individuals’ engagements in cyberbullying (H1–H3). Thirdly, the binary logistic regression was also utilized for constraints, morality, and strain to trace the protective effects on cyberbullying perpetrators and cyber bully-victims, which was expected to find the probability that the constraints and morality can reduce the levels of strain and then decrease the risks of engaging in cyberbullying (H4 and H5). On the first step, the average scores for constraints (mean = 2.48) and morality (mean = 3.09) were used to divide participants into low constraint groups (<2.48), high constraint groups (>2.48), low morality groups (<3.09), and high morality groups (>3.09). On the second step, the sign (positive or negative) of the regression coefficients indicates in what way a predictor variable is related to the outcome variable (Maroof, 2012). In all regressions, an odds ratio is a comparison of an event occurring in two different groups (Maroof, 2012).

## Results

### Descriptive analyses

Descriptive analysis shows that the percentages of cyberbullying perpetrators and victims are 217 (23.40%) and 215 (23.20%), respectively. The prevalence rates of cyberbullying perpetration and victimization are similar to those shown in previous studies (Camerini et al., 2020). Specifically, for cyberbullying perpetration, the top three frequently-used forms were attacking and insulting others (46.30%), breaching others’ privacy (44.90%), and creating fake information (41.70%). For the cyberbullying victimization, more than half of all participants (50.30%) reported having been harassed *via* a large volume of messages, with 46.50% of cyberbullying victims effectively squeezed out of a community, and 46.00% were threatened or intimidated. There were 347 (37.40%) cyber bully–victims; 48.19% of them were threatened or intimidated, and 43.98% of the victims had been squeezed out of a community. Cyber bully-victims’ most often used cyberbullying forms were insulting and attacking others (44.58%), creating fake information, and breaching others’ privacy (each at 43.37%). Besides, there is a significant difference between the years of using social media and cyberbullying roles (*χ*^2^ = 38.23, *p =* 0.00), but surprisingly, we did not find any significant differences between gender (*χ*^2^ = 1.87, *p* = 0.60), age (*χ*^2^ = 9.42, *p* = 0.67), education (*χ*^2^ = 17.57, *p* = 0.12), frequency of using social media (*χ*^2^ = 20.63, *p* = 0.15) and cyberbullying roles, which diverged from findings in previous studies.

### Testing the effects of negative emotion on the strains of cyberbullying roles

The binary logistic regression firstly showed the results amongst negative emotions, strain and cyberbullying roles (cyberbullying perpetration and cyber bully-victims). As shown in Table 1, when participants had a high level of negative emotion, the models were not significant (*p* > 0.05). Therefore, we cannot support Hypothesis 1. At this point, we did another binary logistic regression analysis for the participant who had low levels of negative emotion. The CB1 model (*χ*^2^ = 9.52, *df* = 1, *p* < 0.01) and the CB2 model (*χ*^2^ = 25.95, *df* = 1, *p* < 0.00) were significant. Meanwhile, Nagelkerke *R*^2^ were.05 and.16, respectively, and the percentages of correctly classified cases were 59.50 and 63.80%. Accordingly, we found that strain has effects on cybebullying behavior when participant was at a low level of negative emotion. Specifically, the risk of cyberbullying perpetrators (*B* = 0.65, *p =* 0.00; OR = 1.91, 95% CI: 1.25–2.91) increased by 90.8% for per unit increase in strain, and cyber bully-victims (*B* = 0.99, *p* = 0.00; OR = 2.70, 95% CI: 1.79–4.07) had higher odds (more than 2.7 times as high) of participating in cyberbullying compared with non-victims and pure perpetrators.

**Table 1:** Results of the binary logistic regression analysis of negative emotion, strain and cyberbullying.

Variable		High Levels of Negative Emotion							Low Levels of Negative Emotion						
		B	SE	Wald	*p*	OR	95% CI for OR		B	SE	Wald	*p*	OR	95% CI for OR	
							Lower	Upper						Lower	Upper
Cyberbullying Perpetrator (CB1)	Strain	−0.31	0.33	0.87	0.35	0.74	0.38	1.41	0.65	0.22	8.97	0.00	1.91	1.25	2.91
	Constant	2.20	1.35	2.67	0.10	9.01			−1.26	0.47	7.45	0.01	0.28		
	Model fit	*R* ^2^ = 0.01, *χ*^2^ = 0.90, *df* = 1, *p* > 0.05							*R* ^2^ = 0.05, *χ*^2^ = 9.52, *df* = 1, *p* < 0.01						
	The percentage of correctly classified cases	72.40%							59.50%						
Cyber Bully-victims (CB2)	Strain	0.43	0.32	1.83	0.18	1.54	0.82	2.87	0.99	0.21	22.45	0.00	2.70	1.79	4.07
	Constant	0.20	1.31	0.02	0.88	1.23			−2.43	0.49	24.32	0.00	0.09		
	Model fit	*R* ^2^ = 0.01, *χ*^2^ = 1.77, *df* = 1, *p* > 0.05							*R* ^2^ = 0.16, *χ*^2^ = 25.95, *df* = 1, *p* < 0.01						
	The percentage of correctly classified cases	87.90%							63.80%						

### Testing the effects of low self-control on the strains of cyberbullying roles

With regard to the individuals at low levels of self-control, strain influenced participation in cyberbullying (see Table 2). The overall model was significant (*χ*^2^ = 7.40, *df* = 1, *p* < 0.01; *χ*^2^ = 25.87, *df* = 1, *p* < 0.00), and the model fit was good. In details, strain has significantly positively effects on cyberbullying behavior. The odds of being cyberbullying perpetrators increased by 67.30% for per unit increase in strain (*B* = 0.52, *p* = 0.01; OR = 1.67, 95% CI: 1.14–2.50); and the odds of cyber bully-victims participating in cyberbullying behavior increased by a factor of 2.52 for every unit increase in strain (*B* = 0.92, *p* = 0.00; OR = 2.52, 95% CI: 1.72–3.69). Therefore, we supported H2 and we infer that individuals who were at a low level of self-control were more likely to have higher levels of strain, and were more likely to engage in cyberbullying behavior.

**Table 2:** Results of the binary logistic regression analysis of low self-control, strain, and cyberbullying.

Variable		*B*	SE	Wald	*p*	OR	95% CI for OR	
							Lower	Upper
Cyberbullying Perpetrator (CB1)	Strain	0.52	0.20	6.70	0.01	1.67	1.14	2.50
	Constant	−1.05	0.44	5.88	0.02	0.35		
	Model fit	*R* ^2^ = 0.04, *χ*^2^ = 7.40, *df* = 1, *p* < 0.01						
	The percentage of correctly classified cases	58.80%						
Cyber Bully-victims (CB2)	Strain	0.92	0.20	22.39	0.00	2.52	1.72	3.69
	Constant	−2.26	0.47	23.23	0.00	0.10		
	Model fit	*R* ^2^ = 0.16, *χ*^2^ = 25.87, *df* = 1, *p* < 0.00						
	The percentage of correctly classified cases	64.20%						

Finally, we ran a logistic regression to test the association between strain and cyberbullying. The overall model was significant (*χ*^2^ = 18.87 v.s. *χ*^2^ = 130.50, *df* = 1, *p* < 0.00), and the Nagelkerke *R*^2^ were.07 and.33, respectively. The percentages of correctly classified cases were 65.40 and 77.40%, respectively. We found that strain has significant positive effects on cyberbullying behavior for cyberbullying perpetrators or cyber bully-victims. As shown in Table 3, for the cyberbullying perpetrators, for per unit increase in strain, the risk of cyberbullying perpetration increased by 55.10% (*B* = 0.44, *p* = 0.00; OR = 1.55, 95% CI: 1.26–1.90); for the cyber bully-victims, the probability of their participation in cyberbullying were more than 2.8 times when per unit increase in strain (*B* = 1.06, *p* = 0.00; OR = 2.90, 95% CI: 2.36–3.56). As above, H3a and H3b were supported. Meanwhile, we investigated that the influences of low self-control (OR = 2.52 vs. OR = 1.67) and strain (OR = 2.52 vs. OR = 1.67) were greater on cyber bully-victims than on cyberbullying perpetrators.

**Table 3:** Results of the binary logistic regression analysis of strain and cyberbullying.

Variable		*B*	SE	Wald	*p*	OR	95% CI for OR	
							Lower	Upper
Cyberbullying Perpetrator (CB1)	Strain	0.44	0.11	17.55	0.00	1.55	1.26	1.90
	Constant	−0.82	0.30	7.58	0.01	0.44		
	Model fit	*R* ^2^ = 0.07, *χ*^2^ = 18.87, *df* = 1, *p* < 0.00						
	The percentage of correctly classified cases	65.40%						
Cyber Bully-victims (CB2)	Strain	1.06	0.11	101.90	0.00	2.90	2.36	3.56
	Constant	−2.48	0.33	56.26	0.00	0.08		
	Model fit	*R* ^2^ = 0.33, *χ*^2^ = 130.50, *df* = 1, *p* < 0.00						
	The percentage of correctly classified cases	77.40%						

### Testing the effects of constraints and morality on the strain and cyberbullying behavior

We also considered the protective influences of constraints and morality on strain and cyberbullying. The binary logistic regression analysis showed that strain influenced participation in cyberbullying at different levels of constraints. As shown in Table 4, the models were significant, in which when individuals were at high levels of constraints, strain has significant effects on cyberbullying perpetrators (*χ*^2^ = 13.72, *df* = 1, *p < 0*.00) and cyber bully-victims (*χ*^2^ = 62.36, *df* = 1, *p < 0*.00). Specifically, with per unit the strain increased, the possibilities of perpetrators and bully-victims participating in cyberbullying behavior increased by 58.30% (*B* = 0.46, *p* = 0.00; OR = 1.58, 95% CI: 1.23–2.03) and 2.8 times greater (*B* = 1.03, *p* = 0.00; OR = 2.80, 95% CI: 2.10–3.75), respectively.

**Table 4:** Results of the binary logistic regression analysis of constraints, strain, and cyberbullying.

Variable		High levels of constraint							Low levels of constraints						
		*B*	SE	Wald	*p*	OR	95% CI for OR		*B*	SE	Wald	*p*	OR	95% CI for OR	
							Lower	Upper						Lower	Upper
Cyberbullying Perpetrator (CB1)	Strain	0.46	0.13	12.99	0.00	1.58	1.23	2.03	0.35	0.20	3.03	0.08	1.42	0.96	2.12
	Constant	−1.27	0.37	11.83	0.00	0.28			0.22	0.58	0.14	0.71	1.24		
	Model fit	*R* ^2^ = 0.08, *χ*^2^ = 13.72, *df* = 1, *p* < 0.00							*R* ^2^ = 0.04, *χ*^2^ = 3.21, *df* = 1, *p* > 0.05						
	The percentage of correctly classified cases	58.70%							76.70%						
Cyber Bully-victims (CB2)	Strain	1.03	0.15	48.10	0.00	2.80	2.10	3.75	0.96	0.18	27.88	0.00	2.61	1.83	3.73
	Constant	−3.39	0.49	47.49	0.00	0.03			−0.94	0.55	2.95	0.09	0.39		
	Model fit	*R* ^2^ = 0.34, *χ*^2^ = 62.36, *df* = 1, *p* < 0.00							*R* ^2^ = 0.21, *χ*^2^ = 38.81, *df* = 1, *p* < 0.00						
	The percentage of correctly classified cases	77.10%							89.40%						

Meanwhile, we also examined the relations between strain and cyberbullying when participants had low levels of constraints. The CB1 model was not significant (*p* > 0.05), in which we did not find the significant relations between strain and cyberbullying perpetration. However, the CB2 model was significant (*χ*^2^ = 38.81, *df* = 1, *p* < 0.00), Nagelkerke *R*^2^ was.21, and the percentages of correctly classified cases was 89.4%. Strain has significant positive effects on cyber bully-victims. It recorded an odds ratio of about 2.66 (*B* = 0.96, *p* = 0.00; OR = 2.61, 95% CI: 1.83–3.73). In other words, cyber bully-victims had more than 2.6 times higher odds of cyberbullying participation. Therefore, we cannot fully support H4.

According to these results, we further tested the direct correlations between constrains and strain, finding that constraints have significant influences on strain (see Table 5). Specifically, there is a significant negative correlation between constraints and strain, in which higher constraints were significantly associated with lower levels of strain. This result still showed the possibilities of using constraints as protective factors to reduce strain and indirectly to decrease the risks of participating in cyberbullying.

**Table 5:** Logistic regression analysis between constraints and strain.

Model	Unstandardized coefficient		Standardized coefficient	*t*	Sig.
	*B*	Std. error	Beta		
(Constant)	1.43	0.10		14.07	0.00
Constraints	−0.11	0.03	−0.09	−4.03	0.00
*R* ^2^	0.55				
*F*	569.68				0.00

Furthermore, we used the binary logistic regression analysis to test associations between strain and cyberbullying at different levels of morality. As shown in Table 6, the CB2 model was significant (*χ*^2^ = 6.59, *df* = 1, *p* < 0.05) when participants were at high levels of morality. In detail, cyber bully-victims were more likely to commit cyberbullying when strain increased (*B* = 0.49, *p* = 0.01; OR = 1.63, 95% CI: 1.13–2.35). Meanwhile, both CB1 (*χ*^2^ = 4.42, *df* = 1, *p* < 0.05) and CB2 (*χ*^2^ = 56.92, *df* = 1, *p* < 0.00) models were significant when the participants had low levels of morality. The Nagelkerke *R*^2^ were.03 and.31, in which the model fits were good. We found that cyberbullying perpetrators (*B* = 0.36, *p =* 0.04; OR = 1.44, 95% CI: 1.01–2.06) and cyber bully-victims (*B* = 1.14, *p* = 0.00; OR = 3.12, 95% CI: 2.22–4.38) with low levels of morality were more likely to engage in cyberbullying behavior when the strain increased, partly supporting H5.

**Table 6:** Results of the binary logistic regression analysis of morality, strain, and cyberbullying.

Variable		High levels of morality							Low levels of morality						
		*B*	SE	Wald	*p*	OR	95% CI for OR		*B*	SE	Wald	*p*	OR	95% CI for OR	
							Lower	Upper						Lower	Upper
Cyberbullying Perpetrator (CB1)	Strain	0.17	0.22	0.56	0.45	1.18	0.77	1.82	0.36	0.18	4.00	0.04	1.44	1.01	2.06
	Constant	0.35	0.83	0.18	0.67	1.42			−0.76	0.42	3.37	0.07	0.47		
	Model fit	*R* ^2^ = 0.01, *χ*^2^ = 0.56, *df* = 1, *p* > 0.05							*R* ^2^ = 0.03, *χ*^2^ = 4.42, *df* = 1, *p* < 0.05						
	The percentage of correctly classified cases	72.30%							52.50%						
Cyber Bully-victims (CB2)	Strain	0.49	0.19	6.94	0.01	1.63	1.13	2.35	1.14	0.17	43.06	0.00	3.12	2.22	4.38
	Constant	−0.04	0.71	0.00	0.96	0.97			−2.98	0.46	41.47	0.00	0.05		
	Model fit	*R* ^2^ = 0.04, *χ*^2^ = 6.59, *df* = 1, *p* < 0.05							*R* ^2^ = 0.31, *χ*^2^ = 56.92, *df* = 1, *p* < 0.00						
	The percentage of correctly classified cases	86.30%							70.30%						

## Discussion

Based on GST, the current study examined whether negative emotion and low self-control have influences on cyberbullying behaviors of different roles in China. This study argued that individuals who experienced low negative emotions and low self-control risk engaging in cyberbullying. Specifically, the result of our study showed that low self-control was directly associated with high levels of strain, and, in turn, individuals who were at low levels of self-control were more likely to engage in cyberbullying perpetration or became cyber bully-victims. In addition, we proved that constraints and morality can indirectly reduce strain and decrease the risks of participating in cyberbullying. The current work partially supported GST’s explanatory relevance like many previous studies.

The first step of the current study was investigating cyberbullying in China, finding that cyberbullying behavior is common in contemporary online communication. Overall prevalence rates of pure cyberbullying perpetrators, pure cyberbullying victims, and cyber bully-victims were 23.40, 23.20, and 37.40%, respectively. The relatively consistent proportions of cyberbullying perpetrators and victims might be explained by the participants’ frequent use of technology (Zhou et al., 2013). Unlike instant messaging and email, social media platforms provide a more convenient way of spreading offensive messages. However, we did not find the reason why cyber bully-victims (37.40%) account for the highest percentage of the participants. A possible explanation is that, in the Chinese context, cyberbullying victims often experience offensive communication repeatedly, and the cyberbullying issue may last long. They perpetrate cyberbullying to retaliate for their victimization and regard cyberbullying or other aggression as a means of protecting themselves from severe cyberbullying injuries (Ang, 2016). Meanwhile, the high prevalence of cyberbullying makes people rationalize it “ethically,” which might increase victims’ likelihood of cyberbullying others (Chan et al., 2021). This is consistent with previous findings that cyberbullying victims have the possibilities to engage in bullying behavior (Kwan et al., 2020), but all these might not be the core reasons.

### The roles of strain and cyberbullying

Turning to hypotheses, we did not find the influences of strain on cyberbullying behavior when individuals were at a high level of negative emotion, but interestingly, we found that if individuals had a low level of negative emotion, they may increase the likelihood of participating in cyberbullying when the strain increased. These findings were different from the previous studies, in which, previous studies found that depression and anxiety increased higher possibilities of cyberbullying perpetration (Wang and Ge, 2021; Giumetti et al., 2022). We explained this with three potential reasons. First, the samples from previous studies were mainly about adolescents, but the participants in this study included both youth and adults. At this point, the level of negative emotions may be such a subjective issue and the ability of emotion regulation is also different between adolescents and adults (Young et al., 2019). Researchers have found a clear developmental shifts in how individuals manage emotional responses: emotion regulation capacities developed substantially across adolescence (Gullone et al., 2010; Zimmermann and Iwanski, 2014), and in adulthood, emotional experiences were increasingly effectively managed through internal regulatory strategies (Gross, 2001). Meanwhile, Garofalo and Velotti (2017) explored the relations between difficulties with emotion regulation and aggression, stating that if individuals did not have adaptive ways to deal with the unwanted emotions, aggression might be used as an attempt to externalize these emotions. Therefore, we infer that the capacity to manage one’s own emotional responses determine whether negative emotions can exacerbate the strain and lead to cyberbullying. The difference of the sample elicits different results from existing research.

Secondly, in China, cyberbullying might be a way to express emotion (Xu, 2021), but we did not have evidence to show whether this kind of expression is directly related to strain or the strain caused by negative emotions. For example, when individuals have negative emotions in the real world, they may choose to express themselves online, even using offensive words (Yu and John-Baptiste, 2016). However, there might be many factors (e.g., misinfodemics and quality of life) that led to negative emotions (Pandey et al., 2022). The cyberbullying could be the outcome of negative emotion, but the mediating influence of negative emotions on strain and cyberbullying was unclear in this study.

Thirdly, accompanied by changes in cyberbullying roles, the influences of negative emotions on strain have undergone notable change (Keith, 2018; Lee et al., 2021). For cyberbullying victims, they experienced higher levels of depression and emotional distress than perpetrators and non-victims, and these victims also suffered from more direct, unfair, and serious negative consequences (Lee et al., 2021). Therefore, if cyberbullying victims cannot regulate the negative emotion (e.g., anger and frustration), they were more at risk of engaging in cyberbullying behavior and became perpetrators (Den Hamer and Konijn, 2016). This point is consistent with findings from the Cyclic Process Model and GST (Den Hamer and Konijn, 2016; Wooten, 2021). Yet, although negative emotions may arise possibilities to lead cyberbullying victims being in perpetration (Chu et al., 2018), victims may also regard cyberbullying as ways of protecting themselves rather than an outcome of strain (Qin, 2022).

With regard to the relations amongst self-control, strain and cyberbullying, we found that low self-control significantly affect strain on different cyberbullying roles, which was in line with GST and previous studies (Lianos and McGrath, 2018; Choi et al., 2022). Generally, people with lower self-control had greater strains and were more likely to engage in cyberbullying behavior, especially for the ones who were cyberbullied (Peterson and Densley, 2017). We explain this phenomenon with two reasons. Firstly, cyberspace provides anonymity and immediacy (Ibrahim, 2022). When individuals had a low level of self-control, the cyberspace’s features provide them with more possibilities to bully others online. Secondly, self-control helps individuals to contemplate and pursue long-term consequences rather than seeking short-term immediate pleasure fulfilled by delinquency (Burt, 2020). Therefore, the association between self-control, strain and cyberbullying may be partially derived from how serious cyberbullying was. In other words, since cyberbullying is anonymous, individuals with low self-control may perceive it as a minor or trivial negative behavior, and meanwhile, they may engage in cyberbullying to decrease strain or fulfill pleasure (Lee et al., 2021). Notably, cyberbullying victims suffer from cyberbullying and are surrounded by cyberbullying “peers” who might be more stressful than pure cyberbullying perpetrators and non-victims (Kwan et al., 2020). Under such circumstances, they increase the frequency of engaging in delinquency, even if they have low levels of self-control.

Additionally, strain has direct effects on cyberbullying behavior. We inferred that higher levels of strain directly lead to a desire for cyberbullying behavior, and cyberbullying victims were at increased risk of involving in delinquency. These align with GST and previous studies, and our results extend the existing theory on this relationship by examining both youth and adults samples, as previous studies have largely discussed this issue among adolescent samples, including a study did in China with minors (Cui and To, 2021). In particular, we observed that the strain had a greater influence on cyberbullying victims. In other words, if cyberbullying victims experienced higher strain levels, they were more likely to become cyberbullying perpetrators than non-victims. From the perspective of GST, this phenomenon was consistent with the point that has been emphasized: an individual’s delinquent behavior can be better explained through negative interactions derived from environmental and contextual settings (Lee et al., 2021). In this context, we assume that the likelihood of one’s involvement in cyberbullying depends on the circumstances and personal traits that cause an increase or decrease in strain. For pure cyberbullying perpetrators, strain affected by external environmental factors has a greater impact on their behavior, and for cyberbullying victims, internal factors have greater impact on their behavior. Thus, when cyberbullying perpetrators experienced stronger negative emotions and were under more strain, they were more likely to engage in cyberbullying. In this case, the perpetrators’ self-control had a limited impact on the strain and cyberbullying. Meanwhile, when a cyberbullying victim suffers from stronger bullying consequences and greater strain, the possibility of involving in cyberbullying may increase.

### The response of influences of constraints and morality on cyberbullying

Considering the risk factors’ influences on cyberbullying behaviors, our findings indicated that constraints and morality had indirect functions in decreasing strain and preventing one from engaging in cyberbullying. Therefore, we infer that we can use constraints and morality as elements to prevent cyberbullying.

Surprisingly, regarding constraints, we only found that when participants had low levels of constraints, with an increased strain, the risk of cyberbullying participation among cyber bully-victims increased. The reason might be that strain and cyberbullying behavior are more of subjective emotions and subjective behavioral choices, while constraints are subjective to various internal, informal, and formal control, therefore, both internal and external conditions should be taken into account while assessing the protective effects of constraints on strain and cyberbullying behavior (Cochran, 2015). However, we cannot say constraints do not have indirect influences on strain and cyberbullying. In our study, we found that higher constraints were directly associated with lower levels of strain, so that it might have possibilities to be linked with a lower risk of cyberbullying (Lee et al., 2021). In other words, if the individual exercises self-restraint and can be restrained by external factors (e.g., peer’s and family’s views), they are more likely to behave politely and kindly and consider the consequences of their behavior, rather than pursuing immediate gratification and pleasure or taking cyberbullying when under strain (Choi and Kruis, 2019; Kabiri et al., 2020). Accordingly, the constraint may be an important factor in preventing cyberbullying.

In addition, importantly, this study found significant influences of morality on strain with respect to reducing the prevalence of cyberbullying, which was consistent with the findings of prior studies (Yang et al., 2018; Shadmanfaat et al., 2019). As shown in the major theories of crime and delinquency, researchers considered the direct links between morality and aggression, stating that people with a high level of morality believed that crime was never the right approach; thus, morality was an important protective factor for delinquency (Antonaccio and Tittle, 2008; Wikström and Svensson, 2010; Jang et al., 2014; Brauer and Tittle, 2017). The improvement of our study is that we considered the interactions between different factors, and thus, the protective effects of morality on cyberbullying were different between roles. Firstly, we found that individuals with low levels of morality were more likely to cyberbully. Therefore, there was a direct protective effect of morality on strain and cyberbullying behavior, in which we shall enhance the morality to decrease the cyberbullying behavior.

However, our study also found something to ponder, that is, strain did not have significant effects on cyberbullying behavior when perpetrators have higher levels of morality. This opposes the work of those studies that a higher level of morality leads lower levels of strain and reduce cyberbullying (Lee et al., 2021; Zhang et al., 2021). One explanation for this finding might be the nature of morality and the characteristics of cyberbullying in China. Undoubtedly, individuals with a moral conscience would regard offline crimes as illegal behaviors (Runions and Bak, 2015). In this case, morality prevents them from breaking laws and regulations, and the factors that affect morality might be social norms, legislation, regulation, and education (Perren and Gutzwiller-Helfenfinger, 2012). However, when encountering strain, especially in cyberspace, some people were more likely to lose moral control over behavior. At this moment, individuals’ behavior might be related to personal traits. Therefore, cyberbullying behavior is more related to cyberspace’s features, one’s attitudes toward cyberbullying behavior, and one’s cognitive-behavioral model. For example, in China, many individuals who believed that cyberbullying was a way to express emotions (especially anger) were more likely to perpetrate cyberbullying (Lu et al., 2022). In such cases, individuals focused more on their personal feelings, and the role of morality in regulating behavior was often overlooked. In other words, when people joint activities that were harmful to others to derive personal strain or pursue pleasure, they may rationalize such behavior and minimize harmful consequences. Even a high sense of personal morality had little restraint on harmful behavior when the perpetrator cannot see the harm they inflict on the victim. Accordingly, we shall pay more attention to online delinquency resulted from disregarding or distorting the consequences of one’s actions.

Furthermore, interestingly, a strong sense of morality even exacerbates cyberbullying for cyber bully-victims when the strain increased, as individuals view cyberbullying as a tool of moral judgment or a way to protect themselves. Regarding the cyberbullying issues, individuals often criticize the target based on their moral values, using offensive language, human flesh searches, and falsification. Cyberbullying victims cannot easily escape from cyberbullying and find ways to protect themselves (Marzano, 2022). Consequently, as long as the harmful results of cyberbullying are ignored, minimized, or distorted (Lin, 2013), victims “must” use the same way as a weapon to resist cyberbullying, so that the protective functions of morality on cyberbullying cannot be activated. Accordingly, although morality has possibilities to diminish the effects of strain on cyberbullying participation, we still cannot discount the fact that individuals use their “personal morality” as a standard to cyberbully others. The possibility that the protective mechanism of morality applies to cyberbullying depends on the influence of other internal and external factors.

### Implications for further study

This study explained cyberbullying through GST in China and considered the functions of constraint and morality on preventing cyberbullying. Consequently, the implications of this study are in both theoretical and practical aspects.

#### Theoretical implications

Theoretically, we found that low self-control caused high levels of strain and affected cyberbullying perpetrator and cyber bully-victims, as predicted by Agnew’s theory. We also stated that, given that low self-control incurred differential risks in cyberbullying in multiple roles, cyberbullying victims were the most at-risk group than pure cyberbullying perpetrators. Above all, the current study contributes to the literature on cyberbullying by first demonstrating the empirical link between strain and cyberbullying, concerning GST, and second by providing a theoretical explanation for exploring the influences of risk factors on different cyberbullying roles (perpetrator, victim, and bully-victim). The uniqueness and originality of current study is to consider constraint and morality as protective factors for the relationship between strain and cyberbullying. Nevertheless, in future studies, if we can classify the cyberbullying role precisely through continuous variables, hierarchal regressions are more reliable for testing the research model.

#### Practical implications

Practically, the contemporary society’s open online communication context makes preventing cyberbullying an important priority. Considering the associations between protective factors (constraint and morality) and cyberbullying roles, we suggested that the prevention and intervention of cyberbullying should adopt a cross-sectoral response to cope with strain, to reduce cyberbullying perpetration and to protect cyberbullying victims. For example, education, technology, emotional attention, and legal considerations should work together to promote effective educational and psychological practices. Firstly, the ability of emotion regulation is closely related to cyberbullying prevention. To reduce the risks of cyberbullying, netizens need to learn effective emotion regulation methods, which can help them to view difficult and negative events reasonably, vent dissatisfaction, and relieve strain in the right way.

Secondly, the effects of strain on cyberbullying vary with the constraints, which means an individual’s internal values may decrease the delinquent effect of peers and external environmental context, while high constraints can buffer their motivations for aggressive behavior. Accordingly, online service providers, policymakers, and educators can make joint efforts to create a legitimate and healthy online environment. Specifically, for the online service providers and policymakers, they can enhance the formal rules or policies of cyberbullying, providing a more clear definition about cyberbullying and its forms to let the individual know what they can or cannot do. For educators, especially for the teachers, informal controls on cyberbullying or tips for enhancing individuals’ internal control to decrease levels of strain are very important. For example, educational programs (e.g., promotional clips and storytelling) can be developed to provide youth a deep understanding of cyberbullying.

Thirdly, cyberbullying victims had greater possibilities of bullying others as cyberbullying could be a way to protect themselves when there are no other effective approaches to controlling the situation that they are faced with. At this point, it is very important to establish effective ways or techniques to reduce the negative impacts of cyberbullying on victims. Firstly, we suggest that it is possible to promote moral growth and emotional understanding. For educators, they can provide positive social supports for cyberbullying victims. The ways include, but should not be limited to, improving critical thinking skills to solve problems when confronted with cyberbullying, and effective ways to seek help. In addition, we shall pay more attention on assessing and addressing the nature of cyberbullying victims’ experiences, including their feelings, thoughts, psychological problems, and intentions towards their perpetrators may help identify individuals at risk of cyberbullying behavior and provide suitable interventions. Therefore, both educational and psychological practices should develop programs for cyberbullying victims of solving mental health problems. The programs can include psychological counseling and affection counseling.

Finally, it is important to consider policies and strategies that strengthen factors that affect morality during cyberbullying. This study suggests that when we consider preventing cyberbullying from the perspective of morality, we should focus more on internal and external elements that may also affect morality. For instance, some cyberbullying perpetrators used delinquency or other extreme online aggressive behavior to express their emotions. In such cases, the factors that affect their online behavior could be peers’ or other netizens’ behavior, their cognitive biases towards cyberbullying, and their ignorance of the consequences of cyberbullying. Accordingly, the online environment’s complexity and individual characteristics inhibit morality’s direct impact on preventing cyberbullying. In practice, this study suggests that we should consider providing more resources for explicit rules (e.g., legislation and discipline) and educational programs on cyber ethics, which are expected to enhance individuals’ correct perception and moral values regarding online delinquency.

### Limitations

Although this study presents some strengths, some limitations should be acknowledged. First, a convenience sample that focused on perceptions of cyberbullying in China was used in this study. At this point, although there are some new and important findings that can be used to explain the cyberbullying phenomenon, the generalizability is limited. The findings based on Chinese netizens should be treated with caution in terms of their applicability to the global population. Meanwhile, the CPB and CBV variables used in this study were binary, so it is challenging to use hierarchal regressions or path analysis to test the moderating effects.

Second, the information selected in this study did not include the participants’ job occupation, or economic status. Meanwhile, the age information selected for this study was the wide age range and we did not know the exact age of the participants. This might be why this study did not find any significant relationship between age and cyberbullying roles. Additionally, although most of the CFA indices showed an acceptable model fit, the index of NFI did not meet the standard. This might because of the survey sample size. We may explore the cyberbullying issue with more relevant validations through some more precise demographic information in the future.

The third limitation is the use of GST principles in part of this study, which may affect the comprehensiveness of the results. Meanwhile, the participants in this study did not include anyone under 16 years old. This reduced the representativeness of the sample. Although according to the observation and previous studies, family strains have minor effects on cyberbullying in the Chinese cultural background, we have no direct evidence for their irrelevance. Notably, cyberbullying is a prevalent and serious problem among minors and adolescents. We cannot ignore the effects of family strain on minors and adolescents in the cyberbullying behavior.

Finally, it is undeniable that although this study presented the developmental issues contributing to cyberbullying and implications for preventing it, anti-cyberbullying approaches proposed in this study should be carefully considered to be applicable only to China or can also be used in other parts of the world. The study sample was from some regions of China. Although we found the protective effects of constraints and morality in terms of reducing cyberbullying, there are various differences, such as web accessibility, cultural contexts, and social background, between China and other regions, which may generalize different results.

### Conclusion

This study affirmed that levels of negative emotion and low self-control increase the likelihood of cyberbullying participation, while constraints and morality had protective effects on coping with strain and prevent cyberbullying. These findings were meaningfully for the development of educational and psychological practices to prevent cyberbullying through exercising the interplay between the risk and the protective factors, especially to help those suffering from cyber victimization. The current study also partially tested GST in the Chinese context. Our results support ongoing research in communication, media, psychological and public health fields, which have consistently shown that both risk and protective factors have different influences on strain and cyberbullying behavior in different roles. This means that researchers who are interested in understanding cyberbullying or cyber aggression among individuals or in different context will need to consider both internal and external factors that influence the behavioral intents. The findings shown in this study are helpful for social media platforms, policymakers, and educators to fully consider the personal traits and psychological mechanisms in developing cyberbullying prevention and intervention strategies.

## Data availability statement

The raw data supporting the conclusions of this article will be made available by the authors, without undue reservation.

## Author contributions

WL created the main study framework, collected the data, and wrote the manuscript. HP guided the revision of the study. All authors contributed to this article and approved the submitted version.

## Conflict of interest

The authors declare that the research was conducted in the absence of any commercial or financial relationships that could be construed as a potential conflict of interest.

## Publisher’s note

All claims expressed in this article are solely those of the authors and do not necessarily represent those of their affiliated organizations, or those of the publisher, the editors and the reviewers. Any product that may be evaluated in this article, or claim that may be made by its manufacturer, is not guaranteed or endorsed by the publisher.

## Appendix

### Terms of constraints

Would you feel ashamed of yourself if you engaged in cyberbullying? [internal control].

Would most people whose opinions you value lose respect for you if you engage in cyberbullying? [informal control].

Do you think you would be found by your family, peers, or other people around if you engage in cyberbullying? [formal control].

How much would it matter if you feel ashamed of yourself for engaging in cyberbullying? [internal control].

How serious would it be if most people whose opinions matter to you lose respect for you because you engage in cyberbullying? [informal control].

How serious would it be if you were formally or informally punished for engaging in cyberbullying? [formal control].
